# Bihemispheric Posterior Inferior Cerebellar Artery Occurring with an Azygos Anterior Cerebral Artery: Case Study

**DOI:** 10.1155/2014/541081

**Published:** 2014-05-13

**Authors:** Jamie Toms, Rishi Wadhwa, Sudheer Ambekar, Hugo Cuellar

**Affiliations:** ^1^Department of Neurosurgery, Louisiana State University Health Sciences Center Shreveport, 1501 Kings Highway, Shreveport, LA 71130-3932, USA; ^2^Department of Neurosurgery, University of California, 400 Parnassus Avenue, San Francisco, CA 94122, USA

## Abstract

Variations in intracranial vasculature are well known. We report a rare anatomic variation in a patient who underwent cerebral angiography for suspected intracranial aneurysm. Digital subtraction angiography revealed a bihemispheric posterior inferior cerebellar artery (PICA) and an azygous anterior cerebral artery (ACA). There was no evidence of any aneurysm or vascular abnormality. To our knowledge, this is the first reported case of a patient with a common PICA supplying both the cerebellar hemispheres and a common ACA supplying ACA territory bilaterally. It is important for the physician to be aware of these anatomical variations in order to differentiate a normal variant from a pathological condition.

## 1. Introduction

Anatomical variations are patterns that deviate from the normal anatomical arrangement in the absence of functional impairment. The understanding of such variation is of the utmost importance when the vascular supply to the central nervous system is concerned. Not only understanding the common anatomical differences in vasculature and vascular territories is useful in the diagnosis of peculiar pathological processes, but also this knowledge is imperative when surgical procedures are being performed [1, 2].

Two vessels with known anatomical variations are the posterior inferior cerebellar artery (PICA) and the anterior cerebral artery (ACA). Variations that occur are due to the embryologic development of these vessels [3, 4]. The more common variations of PICA consist of unilateral agenesis and hyperplasia, double or duplicated origins, or epidural or extracranial origin [5–7]. In rare cases, a single enlarged bihemispheric PICA can supply territory in both hemispheres of the cerebellum [5, 6]. Another neurovascular abnormality, where a single artery supplies both sides of the brain [8], is the azygos anterior cerebral artery (ACA). This occurs in only 0.1–0.5% of patients, when there is a fusion between the A2 segments of the right and left ACA and a single vessel supplies both cerebral hemispheres [3, 5, 9].

To our knowledge, this is the first reported case of a patient presenting with both an azygos ACA and a bihemispheric PICA.

## 2. Case Study

A 38-year-old white male presented with a sudden onset of diffuse headaches. On physical examination his vital signs were all within normal limits. He was alert and oriented to person, place, time, and situation with a Glasgow Coma Score of 15. His cranial nerves II through XII were intact. He had strength 5/5 in upper and lower extremities bilaterally, and his sensory examination revealed no deficit. His reflexes were 2+ throughout, and he did not have any pathologic reflexes including Hoffman's, Babinski sign, clonus, or drift. A computed tomographic image without contrast showed no evidence for subarachnoid hemorrhage, but a computed tomographic angiogram (CTA) of the brain showed a possible right anterior cerebral artery aneurysm. To adequately evaluate for possible aneurysm, an angiogram was performed. An 18-gauge angiocath was used to access the right femoral artery; there was selective catheterization of the right and left common carotid arteries, internal carotid arteries, and vertebral arteries.

**Figure 1:**
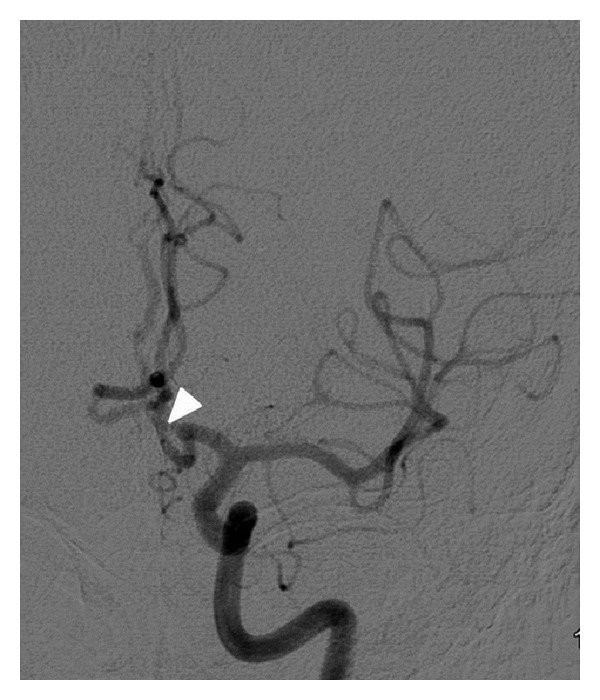
Left internal carotid injection. The arrowhead shows an azygous A2 from which both the pericallosal arteries arise.

**Figure 2:**
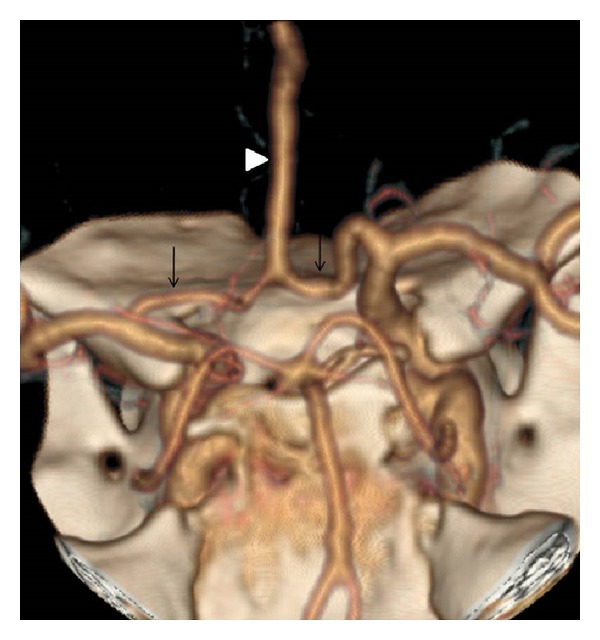
Computed tomographic angiogram showing the azygous A2.

The right internal carotid artery showed a hypoplastic A1 segment, faintly filling the A2 segment; the left internal carotid artery showed a dominant A1 with a common short A2 segment (Figure 1), that bifurcated into the bilateral pericallosal arteries forming the azygos type anterior cerebral artery (Figure 2).

**Figure 3:**
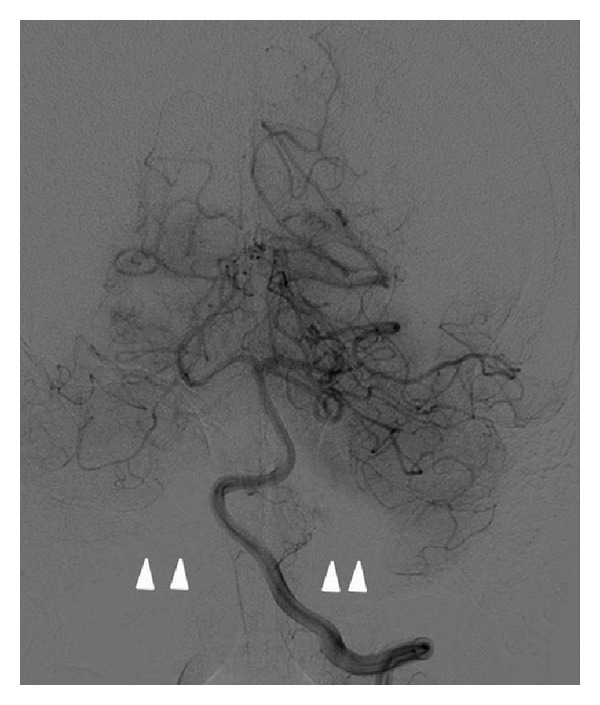
Left vertebral artery injection. There is no filling of the PICA and the corresponding territory.

**Figure 4:**
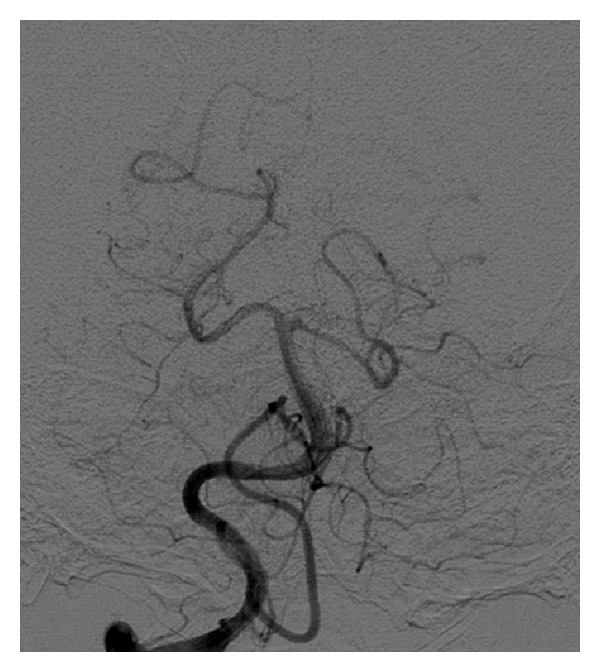
Right vertebral injection. The right PICA gives branches to supply the PICA territory in the left hemisphere.

The right and left vertebral arteries were unremarkable for aneurysm or stenosis, but the left vertebral artery showed agenesis of the left PICA with no opacification of the corresponding cerebellar territory (Figure 3). The PICA branching from the right vertebral artery was enlarged and crossed the midline to supply both cerebellar hemispheres (Figure 4).

## 3. Discussion

Anatomical variation is important to understand especially when considering the vascular supply to the central nervous system. Many alternatives of neurovascular anatomy exist, and it is imperative for surgeons, radiologist, pathologists, and clinicians to have full understanding of the anatomical, pathological, surgical, and embryological implications of such variants. Two such deviations from the norm are the bihemispheric PICA and the azygos ACA.

The path and territory of the posterior inferior cerebellar artery is tortuous and variable [4, 5, 10, 11]. By definition, it is the only cerebellar artery that arises from the vertebral artery [7, 12, 13]; it may also in some cases arise directly from the basilar artery [13]. It travels around the medulla to supply the tonsil, inferior vermis, choroid plexus of the fourth ventricle, and inferior surface of cerebellum [4, 14].

The normal embryological development of the PICA, as described by Macchi et al., represents a recent phylogenic development. At day 30 of estimated ovulation age the cerebellar rudiment becomes present in very close proximity to the fourth ventricle. At this point in development, day 35, the only blood supply to the cerebellum is from the superior cerebellar arteries. It is not until day 44 that the PICA becomes apparent, as a small vessel that terminates in the choroid plexus, and it is not until much later that the definitive path of the PICA is established. This late development of the PICA could explain the large variability in its pathway as well as many of its deviations from the norm [13]. These variations include the more common unilateral agenesis and hypoplasia, double or duplicated PICA origins, and epidural or extracranial PICA origins [5–7] as well as the exceedingly rare bihemispheric PICA [5, 6].

Cullen et al. report an absence of one PICA in up to 26% of patients and also state the most frequent PICA variation is PICA agenesis or hypoplasia. Usually in patients with this variation, the ipsilateral anterior inferior cerebellar artery (AICA) supplies blood to the posterioinferior part of the cerebellum. There have only been a handful of reported cases where a single PICA feeds both cerebellar hemispheres, and although the incidence of this anomaly is unknown [5, 6, 15], a report by Cullen et al. hypothesizes that it occurs in less than 0.1% of individuals [5]. Another report suggests that the anomaly may occur in 3.6% of individuals. This higher incidence would make the vascular anomaly even more important to understand [15].

Unlike vessels of the dura, intradural arteries rarely cross the midline and supply contralateral territories, but there are several proposed reasons for the occurrence of a bihemispheric PICA. One cause of this anomaly is the vessel crossing the midline via the cerebellar vermis to supply the contralateral territory [2, 5]. Another reason for this bihemispheric vessel is that a nonspecific network of vessels may bridge the midline. Lastly, a significantly more novel explanation is that a midline PICA arises to supply both cerebral territories as a result of midline fusion. Similar midline fusion is the reason for the basilar artery, the limbic arch, and the anterior spinal artery [5]. Fusion along the midline is also the explanation for the formation of an azygos ACA [5, 11].

In this case, the bihemispheric PICA was an incidental finding, and this is the standard in similar published cases. However, Gaida-Hommernick et al. report a case in which a bilateral cerebellar infarction is caused by the stenosis of a single PICA supplying both cerebellar hemispheres [10]. The bihemispheric PICA, although unproven, has also been speculated to be present in other acute bilateral cerebellar infarctions [15, 16].

Understanding the complex nature of intradural arteries is essential to diagnosis and treatment of neurovascular pathology. The PICA is the most variable and complex of the cerebellar arteries [7], and detailed understanding of its territory and course is essential to understanding, preventing, and remedying troubles in the posterior fossa [4]. The bihemispheric PICA has only been reported a few times in literature, but we feel it should be considered when encountering stroke subtypes and ischemic syndromes of the posterior fossa [5].

The ACA arises from the internal carotid artery distal to the carotid syphon. It crosses the anterior perforating substance and gives rise to the medial striate vessels. At the longitudinal intercerebral fissure it connects with the contralateral ACA by the anterior communicating artery (AComA). After the AComA, the vessel follows the direction of the corpus callosum genu and parallels its contralateral vessel as it continues over the corpus colossus and terminates at the splenium. The ACA is responsible for the irrigation of oxygen and nutrients to both medial cerebral hemispheres and orbitofrontal portions of the brain [8, 16].

The embryological development of this artery begins early when the internal carotid artery (ICA) reaches the forebrain and divides into the olfactory branch and a posterior branch [11, 16]. The olfactory branch, at day 35, will develop into the ACA with a medial branch that forms the AComA [11]. At portions of normal paralleling ACA it is possible to have different patterns that deviate from the norm. For instance, one such deviation can result in a single ACA feeding bilateral ACA territories. This pattern is called an azygos ACA. In this configuration central branches from the ACA supply the rostrum of the corpus callosum, septum pellucidum, anterior portions of the lentiform nucleus, and the head of the caudate [9, 11]. This malformation is caused by a midline fusion of the proximal paired ACAs or by an extended form of AComA [11].

The azygos ACA has been shown to occur with other congenital vascular anomalies. Cases of arteriovenous malformation and aneurysms have been shown in literature [3]. The azygos ACA has also been shown to be associated with central nervous system malformations, including holoprosencephaly and agenesis of the corpus callosum [17]. Bilateral ACA territory stroke from the occlusion of one vessel is also possible when one vessel is feeding both hemispheres as in the case of an azygos ACA [3, 17]. Despite the frequent association with ACA aneurysm occurring with azygos ACA [8], there has not been significant evidence of increased risk of ischemia or stroke associated with an azygos ACA [17].

The bihemispheric PICA and the azygos ACA present in this patient could have a common phylogenic link. Although there are several explanations for their formation, they both can arise from midline fusion of vessels [5, 11]. This is the same type midline fusion that is seen in normal neurovasculature [5], and although these two vascular anomalies have never been reported together in the same patient, merging of vessels could explain their occurrence.

## 4. Conclusion

In the diagnosis of cerebrovascular pathology the PICA and its branches as well as the ACA are of the utmost importance. Together these two vessels supply a large portion of intracranial territory. As this case points out, multiple rare vascular variants can occur in a single patient. This illustrates that vascular anomalies should be considered when patients present with vascular pathology and before procedures are performed.
